# Opioid use and opioid use disorder in mono and dual-system users of veteran affairs medical centers

**DOI:** 10.3389/fpubh.2023.1148189

**Published:** 2023-04-04

**Authors:** Joseph Goulet, Yan Cheng, William Becker, Cynthia Brandt, Friedhelm Sandbrink, Terri Elizabeth Workman, Phillip Ma, Alexander Libin, Nawar Shara, Christopher Spevak, Joel Kupersmith, Qing Zeng-Treitler

**Affiliations:** ^1^VA Connecticut Healthcare System, West Haven, CT, United States; ^2^Yale School of Medicine, New Haven, CT, United States; ^3^Washington DC VA Medical Center, Washington, DC, United States; ^4^Biomedical Informatics Center, George Washington University, Washington, DC, United States; ^5^MedStar Health, Washington, DC, United States; ^6^Georgetown University School of Medicine, Washington, DC, United States; ^7^Georgetown Howard Universities Center for Clinical and Translational Science, Washington, DC, United States

**Keywords:** opioid, opioid-related disorders, veterans, logistic models, observational study

## Abstract

**Introduction:**

Efforts to achieve opioid guideline concordant care may be undermined when patients access multiple opioid prescription sources. Limited data are available on the impact of dual-system sources of care on receipt of opioid medications.

**Objective:**

We examined whether dual-system use was associated with increased rates of new opioid prescriptions, continued opioid prescriptions and diagnoses of opioid use disorder (OUD). We hypothesized that dual-system use would be associated with increased odds for each outcome.

**Methods:**

This retrospective cohort study was conducted using Veterans Administration (VA) data from two facilities from 2015 to 2019, and included active patients, defined as Veterans who had at least one encounter in a calendar year (2015–2019). Dual-system use was defined as receipt of VA care as well as VA payment for community care (non-VA) services. Mono users were defined as those who only received VA services. There were 77,225 dual-system users, and 442,824 mono users. Outcomes were three binary measures: new opioid prescription, continued opioid prescription (i.e., received an additional opioid prescription), and OUD diagnosis (during the calendar year). We conducted a multivariate logistic regression accounting for the repeated observations on patient and intra-class correlations within patients.

**Results:**

Dual-system users were significantly younger than mono users, more likely to be women, and less likely to report white race. In adjusted models, dual-system users were significantly more likely to receive a new opioid prescription during the observation period [Odds ratio (OR) = 1.85, 95% confidence interval (CI) 1.76–1.93], continue prescriptions (OR = 1.24, CI 1.22–1.27), and to receive an OUD diagnosis (OR = 1.20, CI 1.14–1.27).

**Discussion:**

The prevalence of opioid prescriptions has been declining in the US healthcare systems including VA, yet the prevalence of OUD has not been declining at the same rate. One potential problem is that detailed notes from non-VA visits are not immediately available to VA clinicians, and information about VA care is not readily available to non-VA sources. One implication of our findings is that better health system coordination is needed. Even though care was paid for by the VA and presumably closely monitored, dual-system users were more likely to have new and continued opioid prescriptions.

## 1. Introduction

The opioid overdose epidemic is a public health emergency. According to the Centers for Disease Control and Prevention (1, 2), drug overdoses accounted for more than 700,000 deaths from 1999 to 2017, almost 400,000 of which involved an opioid. Opioid prescriptions peaked around 2012 (2), however opioid-related mortality continued to increase. After reaching a plateau between 2017 and 2019, during the COVID pandemic there has been a sharp increase in opioid mortality (3). Synthetic opioid use (e.g., fentanyl) is largely responsible for the recent surge (4). Nevertheless, current opioid prescribing rates are still higher than those of the 1990s (5). Similar trends have been seen in the Veterans Administration (VA) – the US’ largest integrated health system – where opioid overdoses increased from 2010 to 2016 while VA opioid prescriptions declined (6, 7).

To promote safer opioid use and decrease mortality, health systems including VA have developed opioid prescribing guidelines including recommending providers generally avoid prescribing opioids for the treatment of chronic pain. If prescribed, providers should check prescription drug monitoring databases for evidence of other controlled substances prescriptions from other prescribers and implement opioid risk mitigation strategies (such as urine drug testing, generally avoid dose escalation above certain thresholds, consider issuance of naloxone, and monitor patients for the development of opioid use disorder (OUD)). Systems’ efforts to achieve guideline concordant care may be undermined when patients access multiple opioid prescription sources. Such fragmentation of care is associated with high-risk prescribing practices and OUD (8, 9) which may be exacerbated by a lack of shared information.

VA is in a unique situation because not only are many VA patients’ dual-system users (10), where in addition to VA care, they also access community care, (11) which may be paid for by the VA. Programs expanding VA’s payment of community care include the Veteran Choice Program (VCP), initiated late 2014 and extended and expended in 2017 (12). The VCP program as well as other community care programs were consolidated under the Veterans Community Care Program (VCCP) in 2018 (12). Prior to the introduction of VCP/VCCP, VA rarely reimbursed community care but between 2014 and 2019, almost a quarter of VA enrollees have received authorization to utilize VCP/VCCP. The community care that VA pays for through VCP/VCCP brings an additional layer of responsibility for VA to understand the impact of dual-system use, especially as it may impact opioid safety, yet no prior studies have examined the impact of dual-system use of VA service and VA paid community care on opioid safety outcomes.

To address this gap in the literature, using the electronic health record (EHR) from Washington DC and Baltimore VA Medical Centers, we assembled a cohort of patients from two groups: mono users (active VA patients who did not use VCP/VCCP services) or dual-system users (active VA patients who also used VCP/VCCP services). We analyzed the new opioid prescription, continued opioid prescription, and OUD in these groups to test the hypotheses that: dual-system use is associated with increased new opioid prescription, continued opioid prescription and diagnosis of OUD.

## 2. Methods

### 2.1. Study population and data sources

**Figure 1:**
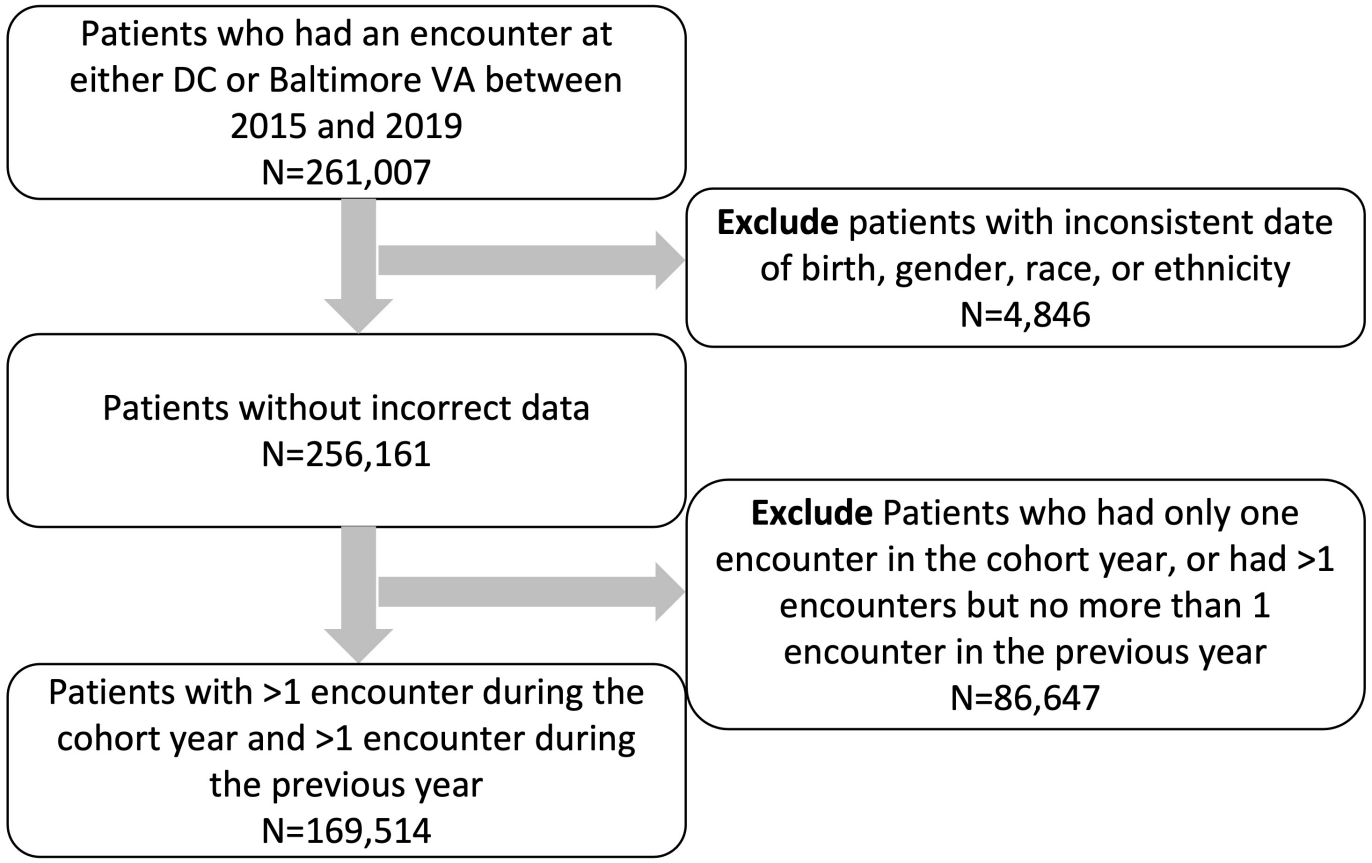
Cohort selection flow chart.

We used VA EHR data from 2015 to 2019 and included active patients, defined as Veterans who had at least one encounter of any type in the VA (in DC or Baltimore) in a calendar year (2015–2019). Each year was treated as separate cohort; active patients in a calendar year with at least one encounter of any type in the prior calendar year were enrolled into the year’s cohort. Additional demographic and clinical information were extracted from VHA electronic clinical and administrative data sources in the Corporate Data Warehouse (CDW) for all eligible Veterans both prior to and following January 1st of each year (index date) to enable the analysis of longitudinal outcomes (13) (Figure 1). The study was approved by the VA IRBnet protocol #1607134.

### 2.2. Use patterns definitions

We defined a VCP/VCCP encounter by use of a VA stop code designated for the community care program or VCP/VCCP note. A VA-only user (“mono”) in a calendar year was defined as a patient without any VCP/VCCP encounter in the calendar year. A dual-system user in a calendar year was defined as a patient with both VA and VCP/VCCP encounters in the calendar year. Each year, fewer than 10 patients had only VCP/VCCP encounters; thus, a VCP/VCCP only group was not warranted.

### 2.3. Study outcomes

Our outcome measures were three binary measures: new opioid prescription, continued opioid prescription (i.e., received an opioid prescription), and diagnosis (during the calendar year). New opioid prescription was defined as no opioid prescription in the VA prior to the opioid prescription in the current calendar year. Continued opioid prescription was defined as receipt of any opioid prescriptions in the current calendar year and had prior prescriptions. Opioid medications included the following: codeine, fentanyl, hydrocodone, hydromorphone, levorphanol, meperidine, morphine, oxycodone, oxymorphone, pentazocine, tapentadol and tramadol. Buprenorphine and methadone were excluded as most of their use is to treat OUD, thus we did not count them as opioids in this specific analysis due to the overlap between their use as pain treatment and as OUD treatment. OUD was defined using the ICD 9/10 codes of 304.00, 304.70, 305.50, and F11.

### 2.4. Covariates

Covariates including demographic and clinical variables were derived from VA data on the index date. We also identified selected comorbid diagnoses recorded at ≥2 outpatient visits or ≥ 1 inpatient stay up to 12 months before entry date. The definition of comorbid diagnoses were defined using the criteria established by the VA MSD cohort (14) and derived from the ICD9/10 codes. Age was measured as a continuous variable and the other covariates were measured as categorical variables.

### 2.5. Statistical analyses

We conducted analyses by year, accounting for the repeated observations on patient and intra-class correlations within patients. We first summarized the baseline characteristics of the dual-system and mono users. We then compared the outcomes between the dual-system and mono user groups.

The main independent variable was the care pattern: dual-system user group vs. mono group. We conducted Chi-square and Student’s *t*-test to compare patient demographics and comorbid conditions between exposure groups as well as outcomes of each group. Due to the large sample size of the study (*N* = 520,049), a very small difference between groups may be statistically significant. Therefore, we also calculated absolute standardized difference (ASD) for each comparison besides reporting value of *p*, for which ASD > 10% indicates imbalanced characteristics between two groups. For each outcome, we conducted a multivariate logistic regression using a dual-system use indicator and all covariates. All analyses were conducted using SAS 9.4. (Cary, NC).

## 3. Results

The number of VCP/VCCP patients increased over time (Table 1). There are total of 169,514 unique patients between 2015 and 2019. We found that the two populations differed on several characteristics. Table 2 shows that the baseline characteristics of mono and dual-system users in DC and Baltimore VA from 2015 to 2019; the test was carried out between the two groups: the dual-system users and the mono users. Significant between differences were observed for each covariate measured.

**Table 1:** Number of dual-system and mono users by year.

Year	Dual-system users (*N* = 77,225)	Mono users (*N* = 442,824)	All (*N* = 520,049)	New opioid	Continued opioid	OUD
2015	1717	99,741	101,458	9,962 (9.82%)	22,933 (22.60%)	2088 (2.06%)
2016	15,504	86,645	102,149	8,901 (8.71%)	20,360 (19.93%)	1933 (1.89%)
2017	18,095	85,166	103,261	7,942 (7.69%)	17,871 (17.31%)	2079 (2.01%)
2018	15,879	88,645	104,524	6,946 (6.65%)	14,880 (14.24%)	2020 (1.93%)
2019	26,030	82,627	108,657	6,281 (5.78%)	12,741 (11.73%)	2054 (1.89%)

**Table 2:** Baseline characteristics of dual-system and mono users.

	Dual-system (*N* = 77,225)		Mono (*N* = 442,824)		Value of *p*	ASD (%)
	Mean/*N*	Std/%	Mean/*N*	Std/%		
Age	58.0	15.8	61.9	16.5	<0.0001	**24**
**Gender**					<0.0001	
Female	18,388	23.8%	58,510	13.2%		**28**
Male	58,837	76.2%	384,314	86.8%		**28**
Race					<0.0001	
White American	28,730	37.2%	199,752	45.1%		**16**
African American	40,382	52.3%	190,083	42.9%		**19**
Others	2040	2.6%	9,136	2.1%		4
Unknown	6,073	7.9%	43,853	9.9%		7
**Ethnicity**					<0.0001	
Non-Hispanics	71,154	92.1%	398,801	90.1%		7
Hispanics	2,672	3.5%	10,520	2.4%		6
Unknown	3,399	4.4%	33,503	7.6%		**13**
**Comorbidity**						
Hypertension	46,637	60.4%	279,261	63.1%	<0.0001	6
Diabetes mellitus	23,589	30.5%	132,833	30.0%	0.0021	1
Post-traumatic stress disorder	23,817	30.8%	92,999	21.0%	<0.0001	**23**
Drug use disorder	29,065	37.6%	155,136	35.0%	<0.0001	5
Alcohol use disorder	16,511	21.4%	83,886	18.9%	<0.0001	6
Tobacco use disorder	22,220	28.8%	122,373	27.6%	<0.0001	3
Other drug disorder	13,106	17.0%	62,209	14.0%	<0.0001	8
Anxiety	27,823	36.0%	113,549	25.6%	<0.0001	**23**
Depression	38,094	49.3%	158,578	35.8%	<0.0001	**28**
Traumatic brain injury	7,403	9.6%	28,838	6.5%	<0.0001	**11**
Neck pain	33,697	43.6%	137,178	31.0%	<0.0001	**26**
Backpain	49,653	64.3%	217,035	49.0%	<0.0001	**31**
Cancer	12,751	16.5%	76,813	17.3%	<0.0001	2
Prior opioid prescription	47,567	61.6%	226,885	51.2%	<0.0001	**21**

ASD > 10% indicates imbalance in a characteristic between the two groups.

In bivariate analyses, a smaller proportion of mono users than dual-system users received new opioid prescription (Table 3). Statistical significance in the tests involving continued opioid prescription and OUD was also witnessed because the tests’ value of *p*s were less than the significance alpha level.

**Table 3:** New opioid prescription, continued opioid prescription, and opioid use disorder in mono and dual users in DC and Baltimore VA from 2015–2019.

	Dual User (*N* = 77,225)		Mono user (*N* = 442,824)		Value of *p*
	*N*	%	*N*	%	
New opioid prescription	9,032	11.7%	31,000	7.0%	<0.0001
Continued opioid prescription	9,032	24.8%	69,621	15.7%	<0.0001
Opioid Misuse	2,169	2.8%	8,005	1.8%	<0.0001

**Figure 2:**
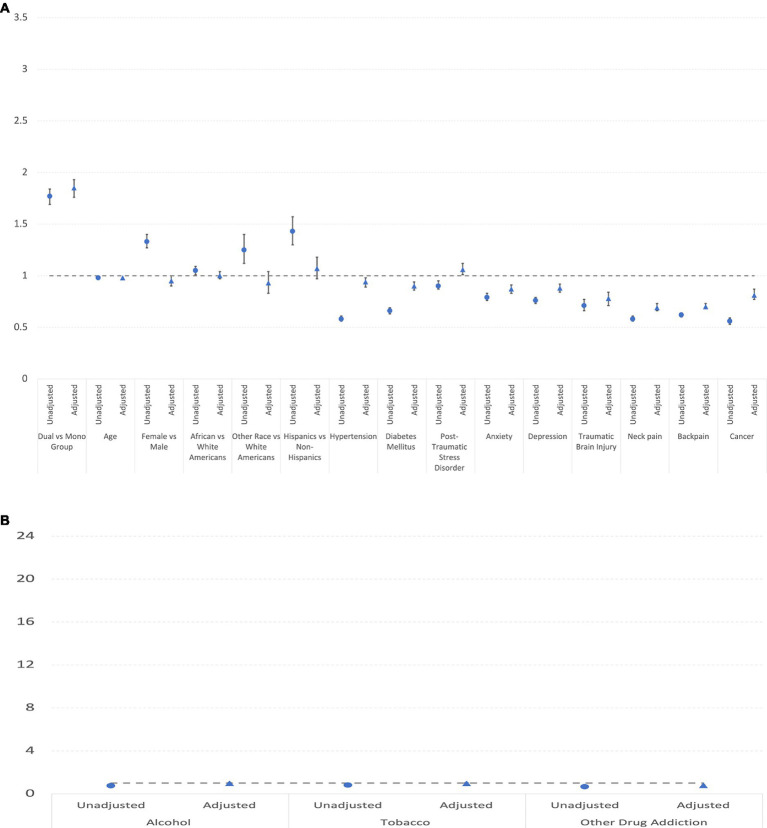
**(A)** Unadjusted and adjusted odds rations and confidence intervals of covariates (excluding addictions) for new opioid prescription. **(B)** Unadjusted and adjusted odds rations and confidence intervals of addiction covariates for new opioid prescription.

Table 4 and Figure 2 presents the results of the analysis of new opioid prescription in mono vs. dual-system users in DC and Baltimore VA from 2015 to 2019. Dual-system users were clearly more likely to have new opioid prescriptions (We separated the addition-related covariates because their odds ratio for the continued opioid prescription and OUD were particularly large). Some comorbidities were statistically significant in the adjusted analyses with OR < 1 because patients with prior conditions such as pain, TBI and cancer often have been receiving opioid and thus does not meet the criteria of new opioid prescription.

**Table 4:** New opioid prescription in mono vs. dual-system users in DC and Baltimore VA from 2015 to 2019.

	Unadjusted OR		Adjusted OR*	
	OR (95% CI)	Value of *p*	OR (95% CI)	Value of *p*
Dual-system vs. mono group	1.77 (1.69, 1.84)	<0.0001	1.85 (1.76, 1.93)	<0.0001
Age	0.98 (0.98, 0.98)	<0.0001	0.98 (0.98, 0.98)	<0.0001
Female vs. male	1.33 (1.27, 1.40)	<0.0001	0.95 (0.90, 1.00)	0.0346
African vs. white Americans	1.05 (1.01, 1.09)	0.0177	1.00 (0.97, 1.04)	0.8442
Other Race vs. white Americans	1.25 (1.12, 1.40)	<0.0001	0.93 (0.83, 1.04)	0.1770
Hispanics vs. non-Hispanics	1.43 (1.30, 1.57)	<0.0001	1.07 (0.97, 1.18)	0.1978
Hypertension	0.58 (0.56, 0.61)	<0.0001	0.94 (0.89, 0.98)	0.0051
Diabetes mellitus	0.66 (0.63, 0.69)	<0.0001	0.90 (0.86, 0.94)	<0.0001
Post-traumatic stress disorder	0.90 (0.87, 0.95)	<0.0001	1.06 (1.01, 1.12)	0.0169
Alcohol	0.75 (0.71, 0.78)	<0.0001	0.98 (0.92, 1.04)	0.4823
Tobacco	0.80 (0.77, 0.84)	<0.0001	0.97 (0.93, 1.02)	0.2898
Other drug addiction	0.66 (0.62, 0.70)	<0.0001	0.78 (0.73, 0.84)	<0.0001
Anxiety	0.79 (0.76, 0.83)	<0.0001	0.87 (0.83, 0.91)	<0.0001
Depression	0.76 (0.73, 0.79)	<0.0001	0.88 (0.84, 0.92)	<0.0001
Traumatic brain injury	0.71 (0.66, 0.77)	<0.0001	0.78 (0.71, 0.84)	<0.0001
Neck pain	0.58 (0.56, 0.61)	<0.0001	0.69 (0.66, 0.73)	<0.0001
Backpain	0.62 (0.60, 0.64)	<0.0001	0.70 (0.68, 0.73)	<0.0001
Cancer	0.56 (0.53, 0.59)	<0.0001	0.81 (0.77, 0.87)	<0.0001

* OR generated using proc genmod with repeated statement.

**Figure 3:**
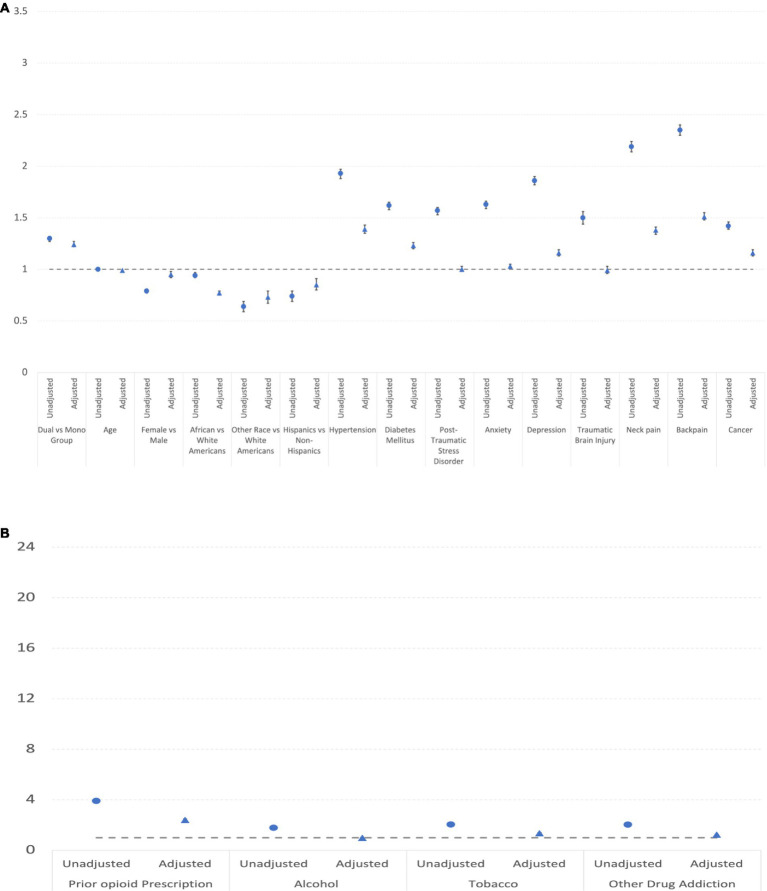
**(A)** Unadjusted and adjusted odds rations and confidence intervals of covariates (excluding addictions) for continued opioid prescription. **(B)** Unadjusted and adjusted odds rations and confidence intervals of addiction covariates for continued opioid prescription.

Table 5 and Figure 3 shows the continued opioid prescription in mono vs. dual-system users in DC and Baltimore VA from 2015 to 2019. There were 18,978 dual-system users, accounting for 24.6% of the dual-system users’ participants and a total of 68,749 mono users, accounting for 15.5% of the mono users’ participants. In the analysis, all variables produced statistically significant effects in both unadjusted and adjusted OR, except the African American vs. white Americans variable in the unadjusted analysis and PTSD and TBI in the adjusted analysis. Unlike in the case of new opioid prescription, comorbid conditions are associated with more continued opioid prescription.

**Table 5:** Continued opioid prescription in mono vs. dual-system users in DC and Baltimore VA from 2015 to 2019.

	Unadjusted OR		Adjusted OR*	
	OR (95% CI)	Value of *p*	OR (95% CI)	Value of *p*
Dual system vs. mono group	1.30 (1.27, 1.32)	<0.0001	1.24 (1.22, 1.27)	<0.0001
Age	1.00 (1.00, 1.00)	<0.0001	0.99 (0.99, 0.99)	<0.0001
Female vs. male	0.79 (0.77, 0.81)	<0.0001	0.95 (0.92, 0.98)	0.0038
African vs. white Americans	0.94 (0.92, 0.97)	<0.0001	0.77 (0.75, 0.79)	<0.0001
Other race vs. white Americans	0.64 (0.59, 0.69)	<0.0001	0.73 (0.67, 0.79)	<0.0001
Hispanics vs. non-Hispanics	0.74 (0.69, 0.79)	<0.0001	0.85 (0.80, 0.91)	<0.0001
Hypertension	1.93 (1.88, 1.97)	<0.0001	1.39 (1.35, 1.43)	<0.0001
Diabetes mellitus	1.62 (1.58, 1.65)	<0.0001	1.23 (1.20, 1.26)	<0.0001
Post-traumatic stress disorder	1.57 (1.53, 1.60)	<0.0001	1.00 (0.98, 1.03)	0.8754
Alcohol	1.77 (1.72, 1.81)	<0.0001	0.98 (0.95, 1.01)	0.2965
Tobacco	2.04 (2.00, 2.09)	<0.0001	1.35 (1.31, 1.38)	<0.0001
Other drug addiction	2.03 (1.98, 2.09)	<0.0001	1.23 (1.18, 1.27)	<0.0001
Anxiety	1.63 (1.59, 1.66)	<0.0001	1.03 (1.00, 1.05)	0.0287
Depression	1.86 (1.82, 1.90)	<0.0001	1.16 (1.13, 1.19)	<0.0001
Traumatic brain injury	1.50 (1.44, 1.56)	<0.0001	0.99 (0.96, 1.03)	0.6641
Neck pain	2.19 (2.14, 2.24)	<0.0001	1.38 (1.34, 1.41)	<0.0001
Backpain	2.35 (2.30, 2.40)	<0.0001	1.51 (1.48, 1.55)	<0.0001
Cancer	1.42 (1.39, 1.46)	<0.0001	1.16 (1.13, 1.19)	<0.0001
Prior opioid prescription	3.91 (3.81, 4.02)	<0.0001	2.40 (2.33, 2.47)	<0.0001

* OR generated using proc genmod with repeated statement.

**Figure 4:**
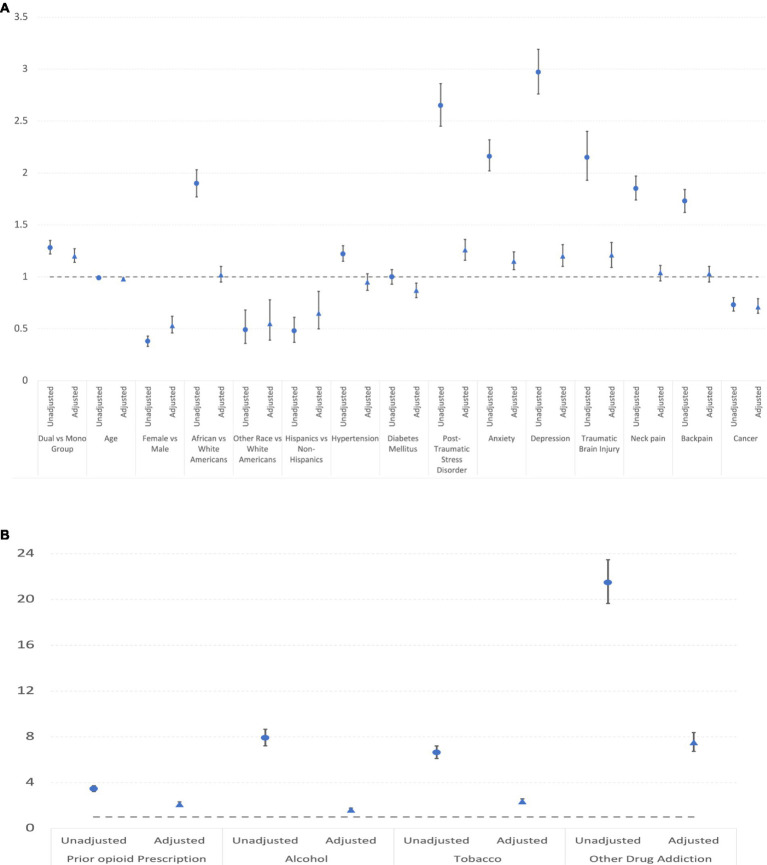
**(A)** Unadjusted and adjusted odds rations and confidence intervals of covariates (excluding addictions) for OUD. **(B)** Unadjusted and adjusted odds rations and confidence intervals of addiction covariates for OUD.

Table 6 and Figure 4 displays OUD in mono vs. dual-system users in DC and Baltimore VA from 2015 to 2019. There were 10,012 dual-system users, accounting for 13% of the dual-system users’ participants and a total of 41,618 mono users, accounting for 9.4% of the mono users’ participants. As with opioid use, a higher risk of OUD is associated with the dual-system user status. Many comorbidities including post-traumatic stress disorder, alcohol and tobacco additional, other addictions, anxiety, depression, and traumatic brain injury significantly increased the OUD. Worth noting is that African American was more likely than white Americans to receive the OUD diagnosis.

**Table 6:** OUD in mono vs. dual-system users in DC and Baltimore VA from 2015 to 2019.

	Unadjusted OR		Adjusted OR*	
	OR (95% CI)	Value of *p*	OR (95% CI)	Value of *p*
Dual-system vs. VA group	1.28 (1.22, 1.35)	<0.0001	1.20 (1.14, 1.27)	<0.0001
Age	0.99 (0.99, 0.99)	<0.0001	0.98 (0.98, 0.99)	<0.0001
Female vs. male	0.38 (0.33, 0.43)	<0.0001	0.53 (0.46, 0.62)	<0.0001
African vs. white Americans	1.90 (1.77, 2.03)	<0.0001	1.02 (0.95, 1.10)	0.5823
Other Race vs. white Americans	0.49 (0.36, 0.68)	<0.0001	0.55 (0.39, 0.78)	0.0007
Hispanics vs. non-Hispanics	0.48 (0.37, 0.61)	<0.0001	0.65 (0.50, 0.86)	0.0027
Hypertension	1.22 (1.15, 1.30)	<0.0001	0.95 (0.87, 1.03)	0.1895
Diabetes mellitus	1.00 (0.93, 1.07)	0.9811	0.87 (0.80, 0.94)	0.0005
Post-traumatic stress disorder	2.65 (2.45, 2.86)	<0.0001	1.26 (1.16, 1.36)	<0.0001
Alcohol addiction	7.90 (7.21, 8.65)	<0.0001	1.61 (1.46, 1.77)	<0.0001
Tobacco addition	6.63 (6.11, 7.20)	<0.0001	2.35 (2.15, 2.58)	<0.0001
Other drugs addiction	21.47 (19.64, 23.47)	<0.0001	7.50 (6.74, 8.36)	<0.0001
Anxiety	2.16 (2.02, 2.32)	<0.0001	1.15 (1.07, 1.24)	0.0002
Depression	2.97 (2.76, 3.19)	<0.0001	1.20 (1.10, 1.31)	<0.0001
Traumatic brain injury	2.15 (1.93, 2.40)	<0.0001	1.21 (1.09, 1.33)	0.0001
Neck pain	1.85 (1.74, 1.97)	<0.0001	1.04 (0.96, 1.11)	0.3388
Backpain	1.73 (1.62, 1.84)	<0.0001	1.03 (0.95, 1.10)	0.5074
Cancer	0.73 (0.67, 0.80)	<0.0001	0.71 (0.65, 0.79)	<0.0001
Prior opioid prescription	3.46 (3.22, 3.72)	<0.0001	2.10 (1.91, 2.31)	<0.0001

* OR generated using proc genmod with repeated statement.

## 4. Discussion

The VA’s implementation of VCP/VCCP has evolved over time, incrementally easing eligibility requirements making it easier for veterans to take advantage of this program (15). In addition, more providers and patients became aware of the VCP/VCCP options. Our study found that dual-system users were younger and have more female and more minority patients. A higher percentage of the dual-system users suffered from depression, anxiety, and PTSD. These characteristics are consistent with the findings of prior reports (16, 17).

Our analysis found that dual-system users receive more opioid prescriptions and exhibit more OUD than mono users after adjusting for demographics factors and comorbid conditions. This is consistent with literature that indicates that patients utilizing dual-system care received more opioid prescriptions (18–20). After adjusting for demographic factors and clinical covariates, the dual-system user status remains significantly associated with a higher risk of new and continued opioid prescription and OUD diagnoses. In addition, the associations of demographic and clinical factors with new opioid prescription initiation and use differed among the VA mono and dual-system user groups in important ways. Younger and female Veterans are slightly less likely to receive opioid prescriptions. Female, Hispanic, and other race Veterans are much less likely to have an OUD diagnosis.

Most comorbidities were associated with a lower risk of receiving new opioid prescription, possibly because many patients with these comorbidities (e.g., cancer) had a pre-existing opioid prescription. Consistent with this hypothesis, we observed that most comorbidities were associated with a higher risk of receiving continued opioid prescription. In terms of OUD, the majority of comorbid conditions were associated with a higher risk; with cancer, diabetes and hypertension being the 3 exceptions. Prior addictions (alcohol, tobacco, and other drug) were all associated with much higher risks of OUD. Their association with new prescription and continued prescription are less consistent. Patients with other drug addictions were less likely to receive new prescriptions but more likely to receive continued prescriptions.

The prevalence of prescriptions has been declining in the US healthcare systems including VA, yet the prevalence of OUD has not been declining at the same rate, with care coordination being a potential issue. A clear implication of our findings is that better system coordination is needed. Cordasco et al. pointed out “Coordinating care is essential for improving patients’ clinical outcomes, enhancing patients’ experiences of care, increasing provider satisfaction, and decreasing costs” (21). Even though VCP/VCCP are paid for by the VA and presumably closely monitored, the dual-system users were more likely to have new or continued opioid prescription. One potential problem is that detailed notes from VCP/VCCP visits are not immediately made available to the VA clinicians and vice versa. Another problem may be that some patients could be intentional “shopping” for providers who are willing to prescribe opioids in the non-VA setting, which is harder to do in the VA. Multiple guidelines have been published for the treatment of chronic pain and opioid dependency (22), but further studies will be needed to understand how system coordination can be improved to reduce the opioid use and OUD in the dual-system use population.

One limitation of this study is that the dual-system user category has been limited to VCP and VCCP. Many veterans have private insurance and VCP/VCCP is only a subset of the dual-system users (23). In addition, OUD is known to be under-documented. The ICD codes we used only can capture some of the OUD. Another limitation to the study is that the decreased prescribing of opioids in medical systems may lead patients to acquire opioids elsewhere. This is more dangerous since there is no quality control on these drugs and the use of fentanyl is now common and is associated with frequent overdoses. In fact, the decrease in opioid prescriptions within the VA (and other systems) could possibly lead to a more dangerous situation for patients for this reason. We did not measure overdoses in this study because overdosed patients are often sent to the nearest emergency department rather than VA. It is however common knowledge that they have increased markedly especially during the pandemic. A decrease in opioid prescriptions does not necessarily mean a corresponding decrease in the number of patients dependent on opioids. We know that the diagnosis of OUD is not an exact reflection of dependence since this diagnosis is often not made in persons who are dependent.

In future studies, we plan to compare the guideline concordant care in both mono and dual-system users. Va and DoD have created clear Clinical Practice Guideline for Opioid therapy (24, 25). There is also ongoing VA efforts to review/oversee opioid prescribing by Community care prescribers to assure safety. This is mandated by MISSION Act 131 and the VA has established a process in all VHA facilities.

We will also perform chart reviews to check if the dual-system users’ medical records lack information on prior history of substance abuse and other risk factors. We would like to carry out a survival analysis. In the survival analysis, each patient will have a single baseline and the mono and dual-system users will be matched at baseline through propensity score matching. In addition, we plan to conduct focus group and interview studies to identify barriers and facilitators of care coordination.

### Resource identification initiative

Data source: heath data from the U.S. Department of Veterans Affairs (RRID:SCR_011566); statistical data analysis software: SAS 9.4 Statistical Analysis System (RRID:SCR_008567).

## Data availability statement

Access to health care data of veterans is legally restricted by the United States Department of Veterans Affairs, which includes both identifying data and sensitive patient information. The VA firewall prohibits the analytic data sets used in this study from leaving without a Data Use Agreement. VA data is accessible to researchers behind the VA firewall with an approved VA study protocol. To learn more about this, please visit https://www.virec.research.va.gov or contact the VA Information Resource Center (VIReC) at vog.av@CeRIV.

## Author contributions

QZ-T and JK designed and supervised the analyses. JG directed the analyses. YC carried out the analyses. TW and AL participated in the data analyses. WB, FS, and CS provided guidance as clinical experts in pain management and opioid use. CB provided guidance on cohort creation. NS facilitated data curation from the EHR. All authors contributed to the article and approved the submitted version.

## Funding

This work is funded by United States Veteran Affairs HSRD IIR grant HX003100-01A2.

## Conflict of interest

The authors declare that the research was conducted in the absence of any commercial or financial relationships that could be construed as a potential conflict of interest.

## Publisher’s note

All claims expressed in this article are solely those of the authors and do not necessarily represent those of their affiliated organizations, or those of the publisher, the editors and the reviewers. Any product that may be evaluated in this article, or claim that may be made by its manufacturer, is not guaranteed or endorsed by the publisher.
